# Challenges to the Modularity Thesis Under the Bayesian Brain Models

**DOI:** 10.3389/fnhum.2019.00353

**Published:** 2019-10-10

**Authors:** Nithin George, Meera Mary Sunny

**Affiliations:** Centre for Cognitive Science, Indian Institute of Technology Gandhinagar, Gandhinagar, India

**Keywords:** modularity hypothesis, attention, precision, cognitive penetrability of perception, predictive coding

## Abstract

Modularity assumption is central to most theoretical and empirical approaches in cognitive science. The Bayesian Brain (BB) models are a class of neuro-computational models that aim to ground perception, cognition, and action under a single computational principle of prediction-error minimization. It is argued that the proposals of BB models contradict the modular nature of mind as the modularity assumption entails computational separation of individual modules. This review examines how BB models address the assumption of modularity. Empirical evidences of top-down influence on early sensory processes is often cited as a case against the modularity thesis. In the modularity thesis, such top-down effects are attributed to attentional modulation of the output of an early impenetrable stage of sensory processing. The attentional-mediation argument defends the modularity thesis. We analyse this argument using the novel conception of attention in the BB models. We attempt to reconcile classical bottom-up vs. top-down dichotomy of information processing, within the information passing scheme of the BB models. Theoretical considerations and empirical findings associated with BB models that address the modularity assumption is reviewed. Further, we examine the modularity of perceptual and motor systems.

## 1. Introduction

The modularity of cognitive processes is a fundamental principle of the representationalist paradigm (Fodor, 1983). Information encapsulation, according to Fodor (2001), is a necessary condition for modularity. It entails the restriction of information flow into a computational module from another module, referred to as cognitive impenetrability. The assumption of information encapsulation was critical to the paradigm shift from the behaviorist to a cognitivist perspective of mental functions as it makes mental processes tractable (Carruthers, 2006) and thus computationally realizable.

Pylyshyn (1980) extended the concept of modularity to computational systems and posited that for any theoretical account of mental process to be explanatory, it should have a cognitively impenetrable functional architecture. For example, the computations in the visual module should have some domain-specific architecture that enables them to transform information uniquely. The range of inputs a module can parse and compute defines domain specificity. Further, Pylyshyn (1980) argued that the modules should be computationally autonomous to make meaningful propositions about mental faculties. A convincing demonstration of information encapsulation in early vision is the persistence of visual illusions, even when one is consciously aware of the illusion. Prinz (2006) counters the argument-from-illusion, by claiming that illusion is an instance of perception trumping belief in the presence of conflict between perception and belief, and when there is no conflict, belief can indeed affect perception. Churchland (1988) argues that visual illusions can be over-ridden by voluntary attempt to modify the character of the visual experience. For instance, when the drawing of a cube lacks visual cues about its orientation, the farther side is interpreted as the closer side by the observer. This illusion, called the Necker-cube illusion, can be overridden by the deliberate “mental” inversion of the cube.

Higher-order cognitive states, such as desires (Balcetis and Dunning, 2006), morality (Gantman and Van Bavel, 2014), and racial category (Levin and Banaji, 2006) is shown to affect perceptual processing. Such instances of top-down effects on perception are argued as evidence for cognitive penetration. In other words, if beliefs affect early visual processes like color perception, it suggests that the information in the “belief processing” module penetrates the color perception module. The strictest form of modularity thesis, known as Massive Modularity, ascribes absolute information encapsulation between all modules of cognition, including the central systems. However, central systems, such as reasoning and decision-making involve the integration of domain-general representations, violating modularity. The defense for massive modularity is that language, with its ability to encode and transform conceptual representations, integrates information across modules (Carruthers, 2006). However, there is empirical support for the notion that content integration is not restricted to faculty of language (Varley and Siegal, 2000), suggesting non-modularity of central systems (Rice, 2011). The modest form of modularity, in contrast, claims that there is a set of common central functions that do not follow information encapsulation. For example, analysis of resting-state BOLD activity has shown the existence of local nodes that are tightly connected within a specific functional module, and connector nodes that integrates information across the individual modules (Bertolero et al., 2015). Modest modularity maintains that input systems, such as perception are modular, whereas, the domain-general integrative processes are non-modular.

Firestone and Scholl (2016) presented an extensive critique of the studies that report top-down effects on early sensory processing. The paper argues that various empirical pitfalls cause the change in perceptual state reported in studies demonstrating top-down effects. Furthermore, the studies that report “valid” top-down effects are explained as peripheral attentional effects. Attention is the mechanism that guides the selection of relevant information from the environment. Attentional mechanisms are found to be responsible for the both enhancement (Carrasco et al., 2000) and inhibition (Tipper, 1985) of sensory representations. The attention-mediation argument for modularity is the proposition that attention affects early perception by selecting one/few representations over others. Consequently, attentional guidance cannot imply penetration as attention is merely changing the output of the early sensory processes. According to Pylyshyn (1999), “[attentional guidance does] not count as cognitive penetration because they do not alter the contents of perception.” Thus, attention is believed to perform the function of integrating the outputs of the impenetrable early sensory processing.

On the contrary, there is another view that the classical models of attention are built on the assumption of modularity, and consequently, attention is not solving the problem of modularity, rather, modularity solves the problem of attention (Van der Heijden, 1995). For instance, the Feature Integration Theory (FIT) (Treisman and Gelade, 1980), a widely accepted model of attention, proposes a dichotomy between bottom-up and top-down processing. In this account, the bottom-up processing involves the computation of fundamental featural dimensions, such as color and orientation by domain-specific units. These bottom-up units are believed to be implementing its natural constraints and are automatic to the extent that it does not engage in an inferential processing (Pylyshyn, 1999). The top-down information, such as goals and desires has no access to these “feature detectors” that processes the fundamental dimensions of the sensory signal. The dichotomy of bottom-up/top-down processing is defined in terms of how each mode of processing is affected by cognitive state. Bottom-up processing is invariant to cognitive states, and top-down processing is influenced by cognitive states. Thus, the evidence that corroborates the proposed distinction between bottom-up and top-down processing (for a review, see Theeuwes, 2010) points to the cognitive impenetrability of the early perceptual processing. Furthermore, the automatic nature of bottom-up units is a defining feature of modular systems (Fodor, 1983). The classical formulation of attention is challenged by the recent models classified as Bayesian Brain (BB) models. In the next section, we review how attention is defined in BB models and place the bottom-up/top-down dichotomy within the information passing scheme of the BB models.

## 2. What Is Bottom-Up in Bayesian Brain?

According to Helmholtz (1925), perception is an inference on the sensory states. This inferential process is necessitated by the absence of one-to-one mapping of the external environment and the information encoded by the senses. Any given sensation could give rise to many possible interpretations. However, we solve what is termed as the “inverse problem” of many-to-one-mapping of the sensory state to internal representation and perceive a relatively stable reality. The inherent ambiguity in the data gathered by the senses necessitates a hypothesis-testing process to build a singular percept (Gregory, 1980). BB models solve the inverse problem by generating optimal prediction about the causes of sensory state. The predictions are compared against incoming information. The information that matches the prediction is “explained away,” and the deviation from the prediction (prediction-error) updates the generative model, which optimizes future predictions (Rao and Ballard, 1999; Friston et al., 2006; Clark, 2013).

BB models posit that the predictive nature of mind entails dissemination of top-down information that affects early stages of perceptual processing. For example, in the version of the BB model developed in Lee and Mumford (2003), the higher-order contextual beliefs interact with early visual processing and the early visual areas are not only doing feature extraction but are also involved in image segmentation and figure-ground segregation. Whether the predictive top-down influence amounts to cognitive penetration is debated. Lupyan (2015) provides an extensive review on how BB models present a case for cognitive penetration of perception based on evidence from cross-modal effects and perceptual illusions. According to Lupyan (2015), the extent of penetrability can be defined in terms of the contribution of a perceptual process toward the minimization of the system level prediction-error (PE).

BB models redefine the classic notions about the nature of bottom-up and top-down information (for a review, see Rauss and Pourtois 2013). According to BB models, the bottom-up information carries prediction-error and top-down information caries predictions about the sensory causes. In the literature, prediction is often referred to as anticipation (Butz and Pezzulo, 2008), expectation (Summerfield and Egner, 2009), preparation (Brunia, 1999). These terms are generally conceptualized in a domain-specific manner in individual studies. Prediction in the BB model involves domain-general signaling about the sensory states and is estimated from the “model-of-the-world.” This generative model encodes the statistical regularities in the environment. The sensory signals that are consistent with the prediction of the generative model are silenced (Summerfield et al., 2008). It is hypothesized that predictions are encoded by the deep pyramidal cells and PEs are encoded in superficial pyramidal cells (Bastos et al., 2012).

According to Free Energy Principle (FEP), which is a generalization of predictive coding, the top-down prediction is weighted by the precision of the PE. Precision quantifies the amount of uncertainty about the information at each level of the cortical hierarchy and is functionally modulated by attention. The metaphor Feldman and Friston (2010) uses for attention is that of Standard Error (SE) in statistical decision-making. The test statistic, on which the statistical inference is made, is obtained by dividing the Mean Difference with the SE. When the SE is high, the test statistic will be low, and thus, the hypothesis is more likely to be rejected. Attention does to perceptual inference what SE does to statistical inference. When implemented as a hierarchical information passing scheme, attention affects perception by optimizing precision. So, signals with higher precision are weighted over signals with low precision. Consequently, at every level in the hierarchy, the signals conveying prediction and attention (precision-weighted PE) information influence perception. In sum, the top-down information is the precision-weighted prediction, referred to as hyper priors (Hohwy et al., 2008) and the bottom-up information is the precision-weighted PE.

The effects of attention observed in studies subscribing to the classical models of attention conflate attention and prediction (Summerfield and Egner, 2009). Hence, the extent to which the effects of prediction and attention are separately contributing to early perceptual effects is mostly unexplored. Empirical evidence corroborating the dissociation of attention, and prediction is demonstrated by orthogonal manipulation of spatial attention and feature prediction. Wyart et al. (2012) found that increase in the prior probability of the occurrence of signal leads to an increase in the baseline performance, whereas the attention cueing lead to increased signal-to-noise-precision at the attended location. Similar manipulation of attention and prediction has shown that attention can reverse the sensory silencing of prediction on BOLD responses (Kok et al., 2011). The difference in BOLD response to expected and unexpected percepts was pronounced in the presence of attention, corroborating the idea that attention improves the precision of PE (Jiang et al., 2013).

O'Callaghan et al. (2017), argued that the top-down effects on early perceptual processing could be considered as penetration by the predictive information, referred to as predictive penetration. This argument is corroborated by neurophysiological evidence reporting the rapid access of top-down information by the early perceptual processing. Orbitofrontal Cortex (OFC) responds to low spatial frequency information of the object ≈ 50 ms before the recognition-related activity started in the Inferior Temporal (IT) area. Early activity in OFC was also better predictive of successful recognition of the object than the activity in the IT region (Bar et al., 2006). Does this suggest that predictions are changing the contents of perception? It is argued that when attention and prediction are separated, the influence of prediction on early sensory processing is restricted to response selection (Rungratsameetaweemana and Serences, 2019). Prediction is found to change the criterion, a signal detection measure of the response bias (Bang and Rahnev, 2017), but not the sensitivity (d′) of the perceptual inference (Summerfield and Egner, 2016). This suggests that prediction alone does not significantly affect early sensory processing.

The bottom-up information, according to the information passing scheme described by the FEP, is not the output of “feature detectors,” but contains the information deviating from the top-down prediction; PE. Empirically, this suggests that classical bottom-up effects are susceptible to the uncertainty (precision) attached to the bottom-up cues. The bottom-up information is continuously modulated by the real-time estimation of precision. Evidence corroborating this has been demonstrated using the irrelevant singleton paradigm (Vatterott and Vecera, 2012), where the attentional capture by color singletons (a classic bottom-up cue) changed as a function of time. Similarly, neural activity associated with “pop-out” like saliency was induced through experience and behavioral relevance (Lee et al., 2002). Observation of experience-dependent changes to classic bottom-up cues shows that precision-weighting dynamically alters early components of perception.

The claim that bottom-up units are not invariant to top-down effects also violates the “automaticity assumption” of modular systems. There have been suggestions that automaticity is conditional on the set of circumstances available to the agent (Bargh, 1989). Anderson and Folk (2014) reported that involuntary response inhibition could be modulated by the mechanisms of goal-directed processing. The Stroop effect was shown to be eliminated when a single letter was colored instead of the whole word (Besner et al., 1997). Future studies investigating precision-dependent changes to classic bottom-up cues can corroborate the information passing scheme proposed by BB models and reconcile the bottom-up/top-down dichotomy.

In the next section, we examine the penetrability of the perceptual systems by the motor systems. Modularity is assumed by many of the influential models that explain perception-action interaction, such as the Optimal Control Theory (OCT) (Wolpert, 1997) and the dorsal-ventral model of vision (Mishkin et al., 1983). We attempt to analyze the modularity assumption within OCT and the alternate proposal by FEP based on a non-modular approach to explain perception-action interaction.

## 3. Modularity of Perception and Action

The separation of perceptual and motor systems as modules that work independently and sequentially is a classic notion in cognitive science. In the classical “sandwich” model (Hurley, 2002), the perceptual system builds the internal representation of the external environment and the motor system derives the motor commands based on the output of the perceptual system, mediated by the integrative cognitive processes. Studies reporting dynamic interaction between action and perception have questioned the classical sandwich model. Estimation of the physical aspects of the environment, such as size, distance, and the slope is found to be modulated by factors, such as effort (Witt et al., 2004), handedness (Linkenauger et al., 2009), graspability (Linkenauger et al., 2011), and skill (Witt and Proffitt, 2005). When participants had to exert more effort to throw a ball at a target, their perceived distance to the target also increased. Objects presented near the hand is also shown to improve perception, manifested as better change detection performance (Tseng et al., 2012), and faster perceptual processing (Thomas and Sunny, 2017).

**Figure 1:**
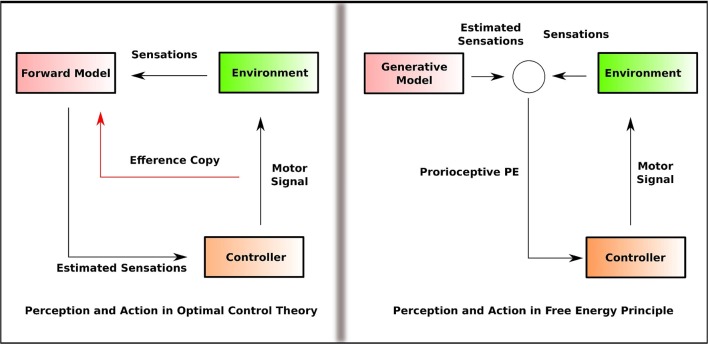
Simplified outline of the information flow in OCT and FEP that illustrates how perception and action are linked. In OCT, perception involves the generation of the predicted sensation that feeds into the controller, and the controller feeds the forward model with the efference copy of the motor command. In FEP, the proprioceptive PE, estimated by comparing the generative model with the sensation, is fed into the controller. Perception and action are separated as forward and inverse model (efference copy) in OCT. In FEP, the inverse model is replaced by the Bayesian inversion of the forward model.

On the one hand, the enactive theories (Varela et al., 2017) posit that such effects can be understood as emerging from the agent-environment interaction, where perception and action are coupled together in a non-modular, non-sequential, and non-encapsulated manner (Baltieri and Buckley, 2018). However, the enactive approach rejects the idea that the agent engages in an inferential process or generate an internal representation of the environment. On the other hand, the optimal control theory of action and motor control assumes that the agent constructs an internal representation of the environment. Importantly, perception and action are considered as separate modules in OCT (Wolpert and Kawato, 1998). In OCT (Wolpert et al., 1995), motor control depends on two computationally independent and informationally encapsulated modules; the estimator and the controller. The estimator predicts the future sensory state based on the current sensory state given the motor command and is referred to as the forward model. The controller provides the motor command that causes the sensory state predicted by the estimator and is referred to as the inverse model. Thus, in OCT, perception, and action are computationally separated as forward and inverse models (Figure 1).

FEP, while maintaining that perception involves inferential processing, proposes a non-modular approach to understand perception-action coupling. Friston (2011) questions the separation of forward and inverse models by OCT and propose an alternative formulation where the Bayesian inversion of the forward model replaces the inverse model. That is, the top-down projections in this framework are not the motor commands, but the predictions about the proprioceptive sensations. PE minimization is achieved in two ways; one, by making accurate predictions about the sensations, two, by acting in such a way that sensations which match the predictions are selectively sampled. This is called active inference (Feldman and Friston, 2010). In active inference, the action minimizes the precision of sensory PE so that the predictions are fulfilled (Friston et al., 2011). This conception views perception and action as inferential processes that are not computationally separated and thus, non-modular. Friston et al. (2010) notes, “the central nervous system is not divided into the motor and sensory systems but is one perceptual inference machine that provides predictions of optimal action, in terms of its expected outcomes.”

FEP combines the non-modular approach of the enactive theories with Helmholzian inferential representations. This representationalist non-modular approach of FEP could be argued as a trivialization of the idea of representation. According to Ramsey (2007), formulating representation as a mediating structure between the external environment and behavior amounts to trivialization. Gładziejewski (2016) argues that the representation in the BB models is as much “action-guiding” as the representation of a cartographic map, and it non-trivially “recapitulates” the causal-probabilistic structure of the environment. Although OCT and FEP maintain a representationalist approach to describe perceptual and motor systems, FEP rejects the separation of forward and inverse models.

The difference between the proposals of the OCT and the FEP can be understood by examining how sensory attenuation of action-effects is explained by both frameworks. Sensory attenuation is the reduction in subjective sensitivity to self-generated sensory-effects. The classic demonstration of this effect is the inability to tickle ourselves (Blakemore et al., 1999). Apart from somatosensation, sensory attenuation of self-caused action-effect has been reported in visual (Cardoso-Leite et al., 2010) and auditory modalities (Hughes and Waszak, 2011). When participants associated a specific sensory outcome (Gabor patch) with a unique action (keypress), the sensitivity to the predicted action-effect was reduced (Cardoso-Leite et al., 2010). According to OCT, the perceived intensity of an action-effect is proportional to the amplitude of the PE (the difference between the forward model prediction and sensation). However, in most of the studies reporting sensory attenuation, the responses are made on the stimuli applied or generated by the agent and not by the experimenter. Voss et al. (2008) observed sensory attenuation for experimenter-generated sensations that occurred while the participant was preparing a movement. This suggests that sensory attenuation happens even when the agent does not generate a forward model (Voss et al., 2008; Brown et al., 2013).

The FEP does not distinguish between the forward and the inverse models. In FEP, sensory attenuation is an effect of reducing the precision of sensory PE. Mechanistically, this is achieved by withdrawing attention from the consequences of action, thereby reducing the intensity of the sensation (Brown et al., 2013). A piece of evidence that points to the role of attention in sensory attenuation was the reduction of action-effect learning when participants were paired with more than one action-effects, suggesting that action-effect association competes for attentional resources (Watson et al., 2015). The presence of valid attention cue is shown to result in faster processing of action-effects (Gozli et al., 2016). In the auditory modality, motor predictions are shown to modulate the action-effect negativity at the posterior electrodes when the stimulus is unattended and not when the stimulus is attended, suggesting an interactive effect of motor prediction and attention on sensory attenuation (Jones et al., 2013). This evidence does not sufficiently corroborate the “withdrawal of attention” hypothesis proposed by FEP. A convincing test of the predictions of FEP about sensory attenuation would be achieved by orthogonally manipulating action-effect prediction and spatial attention to dissociate the separable contribution of action-prediction and attention on sensory attenuation (Schröger et al., 2015a,b).

## 4. Summary

In the current review, we explored the nature of information processing in the BB models and its implications on the assumption of modularity. Recent empirical findings question the classic notion that bottom-up units are invariant to top-down influences. The proposed nature of bottom-up and top-down processing in the BB models is corroborated by empirical findings that report experience-dependent changes to the perceptual quality of the classical bottom-up information. The dynamic and real-time changes in the estimated precision affect perceptual inference. Defining attention as the modulator of precision/synaptic gain provides a rich and nuanced conception of attention.

The early sensory processing is influenced not only by precision-weighting but also by top-down predictions that carry information about expected sensory states. Top-down predictions and bottom-up sensory evidence are affected by attention at each level of the cortical hierarchy. Such a top-down influence is not equivalent to changing the output of early sensation. Clark (2016) claims that the formulation of attention in BB model makes it a mechanism that dynamically re-configures the cognitive architecture of a given stage. Thus, the BB model's definition of attention questions the idea that attention is merely changing the output of early perception. In the context of perception-action interaction, the BB models do not hold a modular view where perception and action are computationally separated as forward and inverse models. The estimator in the FEP minimizes PE by comparing the motor signal with the proprioceptive (sensory) PE, without a separate forward model.

In order to build a unified epistemology of mental functions, the BB models need to explain empirical findings from diverse domains of cognition, emotion, perception, and action. BB models offer a domain-general formulation of information passing, where the external environment and internal representations are defined in terms of their causal-probability structure. In other words, the model itself is neutral about the content of perceptual experience. The perceptual content is determined by the winning hypothesis of the Bayesian inferential process (Hohwy et al., 2008). This definition of perceptual content can facilitate the integration of this framework into the theorizations about diverse mental functions. The attempts to build a unified theory necessitates a novel approach in which the mental function of interest is not ascribed epistemic boundary at the computational level. The BB models appropriate the enactive notion of information flow, where epistemic boundaries between the mind, the body, and the environment are not necessary to explain the behavior of the system.

## Author Contributions

All authors listed have made a substantial, direct and intellectual contribution to the work, and approved it for publication.

### Conflict of Interest

The authors declare that the research was conducted in the absence of any commercial or financial relationships that could be construed as a potential conflict of interest.
